# Phosphorylation-Dependent Intra-Domain Interaction of the Cx37 Carboxyl-Terminus Controls Cell Survival

**DOI:** 10.3390/cancers11020188

**Published:** 2019-02-06

**Authors:** Nicole L. Jacobsen, Tasha K. Pontifex, Paul R. Langlais, Janis M. Burt

**Affiliations:** 1 Department of Physiology, University of Arizona, Tucson, AZ 85724, USA; nljacobs@email.arizona.edu (N.L.J.); tasha@email.arizona.edu (T.K.P.); 2 Department of Medicine, University of Arizona, Tucson, AZ 85724, USA; langlais@deptofmed.arizona.edu

**Keywords:** gap junction, connexin, cell cycle, cell death, gating, phosphorylation

## Abstract

Differential phosphorylation of the carboxyl-terminus of connexin 37 (Cx37-CT) regulates phenotypic switching between cell growth phenotypes (cell death, cell cycle arrest, proliferation). The specific phosphorylation events in the Cx37-CT that are necessary for these growth regulatory effects are currently unknown. Through the combined use of deletion and site specific (de)phospho-mimetic Cx37-CT mutants, our data suggest a phosphorylation-dependent interaction between the mid-tail (aa 273–317) and end-tail (aa 318–333) portions of the Cx37-CT that regulates cell survival. As detected by mass spectrometry, Cx37 was phosphorylated at serines 275, 321, and 328; phosphomimetic mutations of these sites resulted in cell death when expressed in rat insulinoma cells. Alanine substitution at S328, but not at S275 or S321, also triggered cell death. Cx37-S275D uniquely induced the death of only low density, non-contact forming cells, but neither hemichannel open probability nor channel conductance distinguished death-inducing mutants. As channel function is necessary for cell death, together the data suggest that the phosphorylation state of the Cx37-CT controls an intra-domain interaction within the CT that modifies channel function and induces cell death.

## 1. Introduction

Cell death is a key process that plays a pivotal role in different physiological functions, such as organogenesis and cell turnover. However, the dysregulation of connexin (Cx) protein expression, localization, and/or function can contribute to cancer onset, progression, and metastasis; multiple Cx knockout mice were shown to have a significant increase in both spontaneous and chemically induced tumors [1,2,3,4]. Long regarded as tumor suppressor genes, Cxs are a family of proteins that can form dodecameric structures called gap junction channels (GJChs) that enable electrical and metabolic coupling between cells. GJChs are formed by the direct association of two connexons or hemichannels (HChs) from adjacent cells. Cells within a microenvironment can coordinate many cellular processes, including controlled growth, through three main modes of Cx function: (1) intercellular communication via GJChs, (2) transmembrane signaling through HChs, and (3) protein-protein interactions. 

Cx proteins have been implicated in the regulation of cell survival versus death; however, the underlying mechanism(s) remain unclear. The conflicting data in the literature suggest that there is Cx- and cell type-specific regulation of cell survival. Previous reports suggest that the Cx-dependent effects on cell survival require their ability to act as intercellular channels; under pro-apoptotic conditions (e.g., treatment with cytochrome c, heavy ions, or Fas ligand), untreated/healthy neighboring cells also died, suggesting the passage of a “death signal” from cell-to-cell [5,6]. Pharmacologic block of GJChs with non-specific agents reduces the extent of cell death in a variety of cancerous and normal cells [7,8,9,10], further implicating intercellular communication in the propagation of cell death.

It has also been suggested that moderate and/or transient activity of HChs is compatible with physiological function and cell survival, but that massive and/or prolonged opening accelerates cell death [11]. Many Cxs support HCh function under normal [12,13,14] and pathological conditions, such as ischemic injury, metabolic inhibition, and oxidative stress [15,16,17]. Nevertheless, it is generally believed that HChs remain closed most of the time to prevent the loss of key survival factors, such as ATP, NAD^+^, and glutamate, as HCh opening can mediate the extracellular release of these factors and, as such, accelerate cell death.

In contrast to the many studies regarding the regulation of GJCh gating, less is known about HCh open state characteristics. In recent years, studies have indicated that HChs formed by a variety of Cxs close in response to intracellular acidification and extracellular [Ca^2+^]; open probability (P_o_) is greatly reduced with increasing concentrations of H^+^ or Ca^2+^, albeit through sensors presumed to be on opposite faces of the HCh. Like GJChs, P_o_ and the conductance of HChs are also regulated by common post-translational modifications, including phosphorylation. For example, differential phosphorylation of Cx43 in the carboxyl-terminus (CT) has been shown to alter HCh P_o_ and permeability to larger molecules [18,19,20,21]. However, there are currently no available data regarding HCh permeability/selectivity in the regulation of cell death.

It was shown that adenoviral delivery of Cx37, but not Cx40 or Cx43, induces apoptotic cell death in cultured endothelial cells through a channel-dependent mechanism [10]. However, the contribution of GJChs versus HChs to Cx37-induced cell death has not been defined. We showed that Cx37 induces the death of at least some rat insulinoma (Rin) cells, an effect that requires both a functional pore domain and the presence of the CT [22,23,24]. In addition, phosphorylation of the CT regulates P_o_ characteristics of Cx37-based GJChs and HChs; a phospho-mimetic isoform of Cx37 that spends more time in large conductance open states than Cx37-wild type (Cx37-WT) results in substantial apoptotic cell death, whereas HChs formed by dephospho-mimetic Cx37 isoforms, which do not induce death, remain mostly closed.

The current study tests the necessity and sufficiency of different regions of the CT (and thereby phosphorylation sites therein) for Cx37-induced cell death. We hypothesized that Cx37 phospho-isoforms that produce HChs with significant P_o_ and GJChs that close briefly while mainly transitioning between larger conductance states predict cell death. We show that phosphorylation occurs at select serine and threonine residues in the Cx37-CT, many of which are putative targets of growth factor activated kinases. In addition, the data included herein suggest that phospho-specific cooperativity between the end-tail and mid-tail portions of Cx37-CT controls cell survival. Finally, while channel function is necessary for Cx37-mediated cell death, neither HCh conductance nor P_o_, alone, is predictive of the death phenotype. These results support the conclusion that Cx37 regulates the growth phenotype in a phosphorylation-site specific manner and suggest that intra-molecular interactions, and likely intermolecular interactions, are modulated by differential phosphorylation in the CT domain.

## 2. Results

We previously showed that a functional channel and the CT are both necessary, and neither is sufficient without the other, for Cx37-mediated growth suppression [23,24]. Previous data also suggested that channel properties are regulated by specific phosphorylation in the CT in a manner predictive of Cx37’s unique ability to support multiple growth phenotypes of cells (cell death, cell cycle arrest, or proliferation; [22]). For example, a multiphosphosite-mimetic isoform of Cx37 (Cx37-S_7_D_7_) supported the increased P_o_ of large conductance HChs and GJChs that appeared to predict cell death. However, the specific site(s) of phosphorylation in Cx37 regulating cell survival remain unclear.

### 2.1. Intra-Domain Interaction within the Cx37-CT Supports Cell Survival

**Figure 1:**
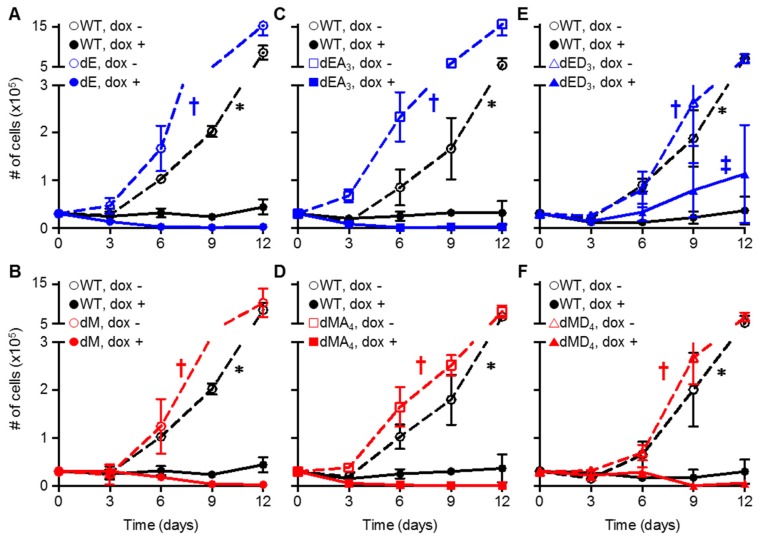
Both the end-tail and mid-tail regions of the Cx37-CT are necessary and mimicking phosphorylation at S275, S285, and S302 in the Cx37-dE mutant is sufficient for cell survival. Proliferation assays revealed that expression of Cx37-WT (black) initiated death of some cells (days 1–3) and an extended period of growth arrest (days 4–12) of the remaining cells following induced expression (dox +) on day 0. Exponential proliferation was evident in non-expressing (dox -) Rin cells. However, expression of Cx37 with deletions of either the end-tail (dE, blue) or mid-tail (dM, red) alone (**A**,**B**), or in combination with alanine substitutions at the remaining putative phosphorylation sites (**C**,**D**; dEA_3_ and dMA_4_), resulted in death of most, if not all, cells. (**E**) Aspartate substitution at S275, S285, and S302 with an end-tail deletion (dED_3_) greatly reduced Cx37-dependent cell death and shortened the growth arrest period such that cells began to slowly proliferate after three days of induced expression. Cx37-dED_3_ cell cycle time between days 6–12: dox -, 1.93 days; dox +, 3.36 days. (**F**) Aspartate for serine substitution at 319, 321, 325, and 328 with mid-tail deletion (dMD_4_) retained the death-inducing properties of Cx37-dM. After 12 days of induced expression, the number of iRin37-dED_3_ cells was significantly different than the number of -dE and -dEA_3_ cells. There was no difference in the number of iRin37-dM, -dMA_4_, and -dMD_4_ cells. *n* = 3 in triplicate for all Cx37-isoforms. All values are mean ± s.e.m (where error bars are not evident, they are smaller than the symbol size). ‡ indicates *p* < 0.05 Cx37-dE versus -dED_3_, non-parametric ANOVA and Kruskal-Wallis multiple comparisons test. *p* < 0.05 for dox + versus dox − for all mutants (†), as well as WT (∗).

As a screening tool to narrow down which putative phosphorylation sites targeted in the Cx37-S_7_D_7_ mutant compromise Rin cell survival, we first deleted either the end-tail (Cx37-dE; aa 318–333) or mid-tail (Cx37-dM; aa 273–317) portions of the Cx37-CT. We transfected otherwise communication-deficient Rin cells with the Cx37-dE or -dM sequences and selected antibiotic resistant clonal cell lines that express within the range demonstrated to be effective for Cx37-mediated growth effects [25]. Despite eliminating large portions of the Cx37-CT, Cx37-dE, and -dM localized to appositional membranes to form functional GJChs, junctional conductance was significantly lower in iRin37-dE cell pairs (Cx37-WT: 3.38 ± 0.7 nS, *N* = 30; -dE: 1.62 ± 0.3 nS, *N* = 26, *p* = 0.03 versus -WT), but iRin37-dM cell pairs were comparable to iRin37-WT (Cx37-dM: 5.01 ± 1.5 nS, *N* = 24). Interestingly, unlike the complete removal of aa 273–333 (Cx37-273tr; [26]), the expression of Cx37 with deletions of only the end-tail or mid-tail region resulted in substantial cell death (Figure 1A,B). This suggests that cross-talk between the end-tail and mid-tail regions is critical in regulating the cell growth phenotype as neither region is sufficient for cell survival without the other.

We next determined whether mimicking phosphorylation or dephosphorylation in the end-tail or mid-tail regions of the Cx37-CT modulated Cx37-dE or -dM-induced cell death. Alanine for serine substitutions, preventing phosphorylation at the remaining putative phosphorylation sites, amplified Cx37-dE and -dM-induced cell death (Figure 1C,D). In contrast, Cx37-dED_3_ (end-tail deletion with aspartate substitutions at S275, S285, S302) attenuated Cx37-mediated cell death, whereas Cx37-dMD_4_ with aspartate substitutions at S319, S321, S325, and S328 retained the death-inducing properties of Cx37-dM (Figure 1E,F). As such, the growth arrest period of Cx37-dED_3_ expressing cells was shortened by six days and iRin37-dED_3_ cells began to slowly proliferate after three days of expression (doubling time: dox −, 1.8 days; dox +, 2.4 days). Using non-parametric ANOVA analysis of cell number across the 12-day period and the Kruskal-Wallis multiple comparisons test, Cx37-dED_3_ was significantly different from -dE and -dEA_3_. Similar comparisons for the Cx37-dM, -dMA_4_, and -dMD_4_ mutants revealed no differences between them. Together, the data suggest that the phosphorylation-dependent interaction between the end-tail and mid-tail regions of the Cx37-CT regulates cell survival.

### 2.2. Cx37 Is a Multi-Phosphorylated Protein

To ascertain which of the seven previously studied [22] high probability site(s) in the end-tail and mid-tail regions might actually be targeted for phosphorylation, we used mass spectrometry to explore Cx37 phosphorylation. Our strategy took advantage of our previously published observation that Cx37-WT is growth suppressive at a low and high cell density, inducing growth arrest in the majority of cells (with cell death as a secondary phenotype) within 24 h of protein expression [25]. Thus, we grew cells to high density; added dox to induce Cx37 expression; and isolated the protein from untreated, and either bisindolylmaleamide (BIM) or phorbol ester (TPA) treated, cells. Cx37 immunoprecipitates were separated by SDS-PAGE and gel slices corresponding to the size of Cx37 were excised, subjected to trypsin digestion, and analyzed by mass spectrometry, as described in Materials and Methods. No quantitative differences in any of the detected Cx37 phosphorylation sites were observed between treatment groups. Reported here are the number of times a Cx37 peptide that contained a putative phosphorylation site was detected and the number of times that the Cx37 peptide was detected as being phosphorylated at a predicted site, irrespective of treatment (Table 1). It is worth noting that other sites in Cx37 were also detected as being phosphorylated, albeit less frequently, including T154, T280, T287, T311, S286 (with T287), S326, and Y177. The kinases predicted with the greatest probability by NetPhos 3.1 to target these sites are cdc2 (T154, 54.5%; T287, 47.8%), CaM-II (T280, 45.9%), PKG (T311, 55.8%), PKC (S286, 51.3%), and INSR (Y177, 49.1%). Never detected as phosphorylated was T301, despite being detected more than 500 times. Additionally, Y332 was never detected as being phosphorylated, consistent with other reports of very little, if any, tyrosine phosphorylation of Cx37 [27,28]. 

Since the predominant phenotype of cells from which Cx37 was harvested was growth arrested, we associated phosphorylation at S319 with the growth arrest versus proliferative phenotype (and preliminary data with alanine/aspartate substitutions at this site support this association). If the remaining lower-incidence sites, when phosphorylated, induce cell death, they would not be expected to be detected as phosphorylated very frequently in samples where the predominant phenotype is growth arrest. 

### 2.3. Phosphomimetic Mutations at Cx37-S321, S328, and S275 Induce Death of Rin Cells

**Figure 2:**
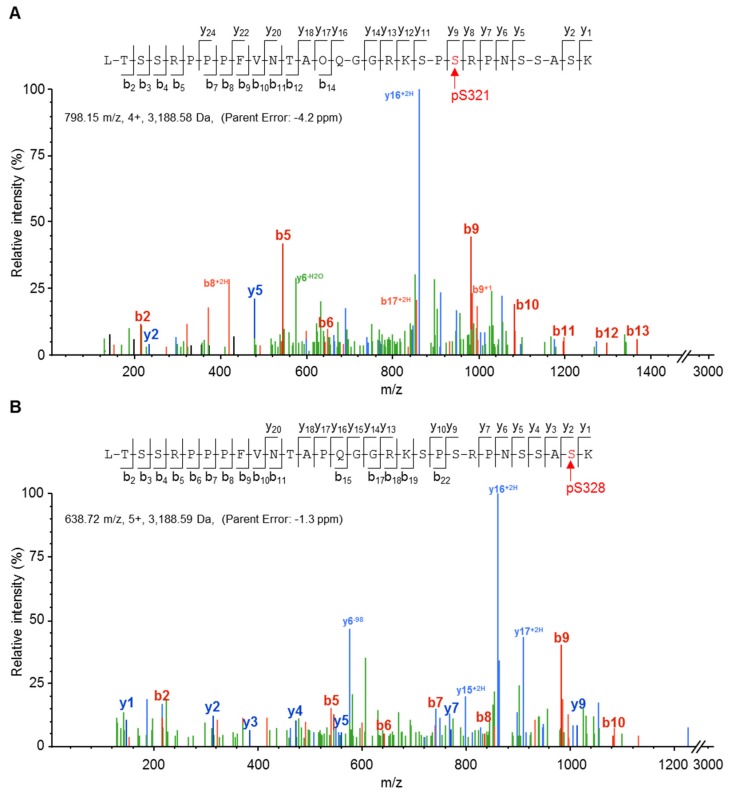
Amino acid residues Ser-321 and Ser-328 of mCx37 are phosphorylated in Rin cells. Representative MS/MS spectra identifying phosphorylation at residue 321 (**A**) and residue 328 (**B**). The observed b-fragment (N-terminal) and y-fragment (C-terminal) ions allowed unambiguous localization of phosphorylation at S321 (**A**) and S328 (**B**), respectively.

The observation of occasional Cx37 phosphorylation at residues S321 and S328 in the end-tail region prompted us to explore the phenotypic effect of single site mutations at these sites. Figure 2 shows that these residues were unambiguously identified as phosphorylated by mass spectrometry.

Given the mass spectrometry data, we investigated the phenotypic consequences of expressing (de)phosphomimetic point mutations at Cx37-S321 and S328 within the end-tail region. As previously mentioned, we hypothesized that the phosphorylation of Cx37-S321 and/or -S328 induces cell death, and that aspartate substitution at either of these sites would result in cell death, similar to the expression of Cx37-S_7_D_7_ [22]. Aspartate for serine substitutions were made at residues 321 or 328 and expressed in iRin cells. Clonal cell lines were selected for further study, with a comparable Cx37-mutant expression level to -WT. The mutant protein trafficked like Cx37-WT to the membrane, where it formed gap junctions; junctional conductance was significantly higher between cell pairs expressing either Cx37-S321D or -S328D versus -WT (Cx37-WT: 3.38 ± 0.7 nS, *N* = 30; -S321D: 7.41 ± 2.2, *n* = 11, *p* = 0.02; -S328D: 7.94 ± 1.1, *n* = 21, *p* = 0.001).

**Figure 3:**
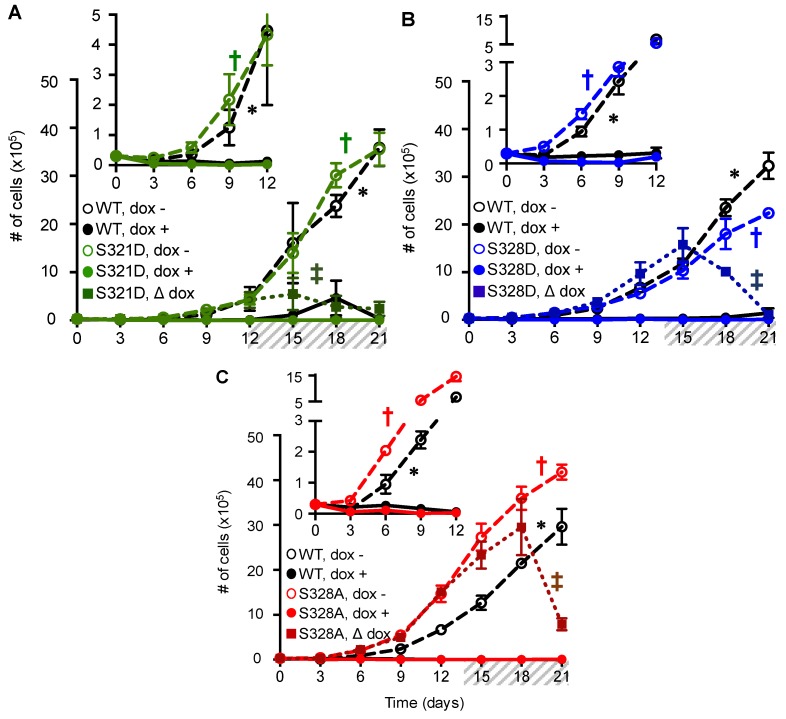
Isoforms mimicking the (de)phosphorylated state of S321 and S328 induce cell death, irrespective of cell-cell contact. Expression of (**A**) Cx37-S321D (green), (**B**) Cx37-S328D (blue), and (**C**) Cx37-S328A (red) induces cell death at low and high density. Insets highlight growth effects in cell cultures with few cell-cell contacts. dox -, no protein expression; dox +, induced Cx37 expression; Δ dox, cells grown to a higher cell density in the absence of dox for 12–14 days, after which dox was included in the media to induce expression, denoted by grey hash. *n* = 3 in triplicate for each isoform and dox treatment protocol. *p* < 0.05 for dox + versus dox − for all mutants (†), as well as WT (∗). For dox-switch experiments, ‡ indicates *p* < 0.05 for growth rate before versus after dox addition. All values are mean ± s.e.m (where error bars are not evident, they are smaller than the symbol size).

In addition to the visual inspection of cells, proliferation assays revealed that the expression (dox +) of either Cx37-S321D or -S328D induced the death of cells, regardless of cell-cell contact (i.e., in both low and high density cell cultures) (Figure 3A,B). Death-inducing properties were particularly evident in the Δ dox condition, wherein cells grew to a higher density (see Figure S1) in the absence of Cx37 expression, after which dox was added to initiate protein expression. For example, 12 days after plating, induced expression of Cx37-S321D reduced the number of cells from 5.5 × 10^5^ at day 15 to 2.8 × 10^5^ and 2.4 × 10^5^ at days 18 and 21, respectively. The magnitude of cell death was even more pronounced in contact-forming iRin37-S328D cells; within seven days of induced expression, the number of cells decreased by more than 10-fold (day 15: 1.58 × 10^6^ cells, day 21: 1.06 × 10^5^ cells). Unexpectedly, the expression of Cx37-S328A also resulted in significant cell death (Figure 3C), an effect not seen in iRin37-S321A cells (Figure S2A). The time required to induce significant cell death was different between these Cx37-mutants, which may reflect the sequence/timing of phosphorylation of each of these serine residues (a possibility not explored in the current study) or differences in the mechanism of induced death. Since apoptosis contributed to Cx37-S_7_D_7_-induced death, we screened these mutants for the activation of caspase 3. Activated caspase 3 was readily detected in non-adherent iRin37-S321D cells, at apparently lower levels in -S328D cells, and still lower levels in -S328A cells (see Figure S3). 

**Figure 4:**
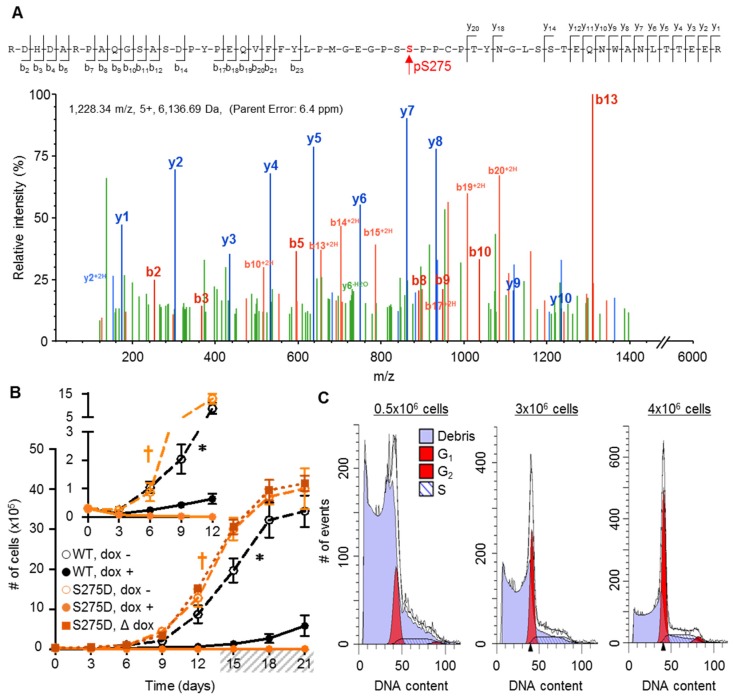
Phospho-mimetic isoform, Cx37-S275D, triggers cell death in a density-dependent manner. (**A**) Representative MS/MS spectra identifying phosphorylation at serine 275 in the mid-tail region of Cx37. Assignment of the indicated ion fragments is shown. Although they show that other possible phosphorylation targets were not phosphorylated, they do not discriminate between S274 vs. S275 as the phosphorylated residue. (**B**) At low density, expression of Cx37-S275D (orange) results in cell death – see in particular the inset. But when expression was induced at higher cell density, where cell-cell contact occurred, no cell death was evident. *n* = 4 for WT, *n* = 3 for S275D for each dox condition. dox -, no protein expression; dox +, induced Cx37 expression; Δ dox, non-expressing iRin37-S275D cells grew to a higher cell density for 14 days, after which dox was included in the media to induce expression, denoted by grey hash. *p* < 0.05 for dox + versus dox − for -S275D (†), as well as -WT (∗). For the dox switch experiment, growth rate before versus after addition of dox was not different. All values are mean ± s.e.m (where error bars are not evident, they are smaller than the symbol size). (**C**) As assessed by propidium iodide DNA staining and FACS, an increase in the number of initially plated cells per 100 mm dish (indicated at the top of each FACS graph), and therefore cell density, reduced the extent of Cx37-S275D-induced cell death. % dead cells in 10^4^ analyzed events: 0.5 × 10^6^ cells, 88%; 3 × 10^6^ cells, 72%; 4 × 10^6^ cells, 55%. Arrowheads on x axis denote the G_0_/G_1_ peak center as identified by the Modfit LT program when possible. Otherwise, peak centers were user defined.

S275 was identified as the phosphorylated residue in mid-tail peptides (Figure 4A) during Mascot/Scaffold analysis of the mass spectrometry data; however, after manual validation of the fragment spectra, we were unable to find critical ion-fragments to distinguish between phosphorylation at S275 versus S274. Consequently, we propose that the assignment of phosphorylation between these sites is ambiguous. Nevertheless, other sites within this large peptide were unambiguously ruled out as the phosphorylated residue by the detected ion fragments (residues: Y259, 266, 281; S254, 256, 285, 286; and T280, 287, 295, 296). Considering that S274/S275 sit at the center of a possible “master regulatory domain” (aa: 273–281), an intrinsically disordered region of the CT containing predicted/documented phosphorylation sites, potential ability to undergo cis-trans proline isomerization, and multiple binding motifs for protein partners [29], these residues are ideally suited to potentially regulate Cx37-mediated growth effects. Comparison of the NetPhos 3.1 predictions for phosphorylation at S274 versus S275 revealed a less than 50% probability for S274 and a greater than 90% probability of phosphorylation at S275. For these reasons, we focused on the role that S275 might play in Cx37-mediated growth regulation.

Considering that iRin37-dED_3_ cells proliferate with minimal/no cell death, we hypothesized that Cx37-S275D would also not induce Cx37-dependent cell death; the corollary here is that phosphorylation within the end-tail region (e.g., S321 and/or S328) regulates cell death, but that Cx37-pS275 may mitigate Cx37-mediated cell death. Interestingly, the expression of Cx37-S275D, but not -S275A (Figure S2B), also resulted in excessive Rin cell death, but in a density-dependent manner (Figure 4B). In low density cells, the induced expression of Cx37-S275D triggered cell death (# of cells day 0: 3 × 10^4^; day 3: 7.34 × 10^3^, day 6: 3.67 × 10^3^) and by day 12, there were no live cells remaining (Figure 4B, inset). In contrast, when Cx37-S275D expression was induced in higher density, contact-forming cells at day 14, cells continued to proliferate exponentially without any impact on cell survival/proliferation, despite robust protein expression. This density dependence of induced cell death was not observed for the other mutants presented herein or for Cx37-S_7_D_7_ [22]. Fluorescence activated cell sorting (FACS) and propidium iodide staining further supported the density-dependent effects of Cx37-S275D (Figure 4C). Propidium iodide was used as a direct measure of DNA content such that cells containing 2n DNA correspond to cells in the G_0_/G_1_ phases of the cell cycle. Therefore, cells (events) containing less than a full complement of DNA are considered dead. Increasing the number of cells initially plated (indicated at the top of each histogram) decreased the percentage of dead cells after six days of Cx37-S275D expression; in an equal number of analyzed cells, the percentage of dead iRin37-S275D cells decreased from 88% to 55% in 0.5 × 10^6^ cells and 4 × 10^6^ plated cells, respectively. Attenuation of Cx37-S275D-induced cell death at high density suggests that HChs and GJChs may have differential roles in Cx37-mediated cell death.

### 2.4. Neither Cx37 HCh Conductance nor P_o_ Is Predictive of Cell Death

Previous data indicated that phospho-isoforms of Cx37 that supported an increased P_o_ of large conductance HChs and/or GJChs correlated with the magnitude of the cell death response. Here, we explored the HCh function of each death-inducing mutant 24–48 h after inducing expression in low density cells using a whole-cell voltage clamp and +25 mV membrane potential, a voltage compatible with the opening of Cx37-WT HChs in isolated cells [30]. The incidence of HCh activity was comparable between death-inducing single site Cx37-mutants and Cx37-WT, but lower than -S_7_D_7_: S321D, 42% *n* = 48; S328D, 50% *n* = 18; S328A, 55% *n* = 29; S275D, 51% *n* = 70; WT, 44% *n* = 27 and S_7_D_7_, 73% *n* = 26 [22].

**Figure 5:**
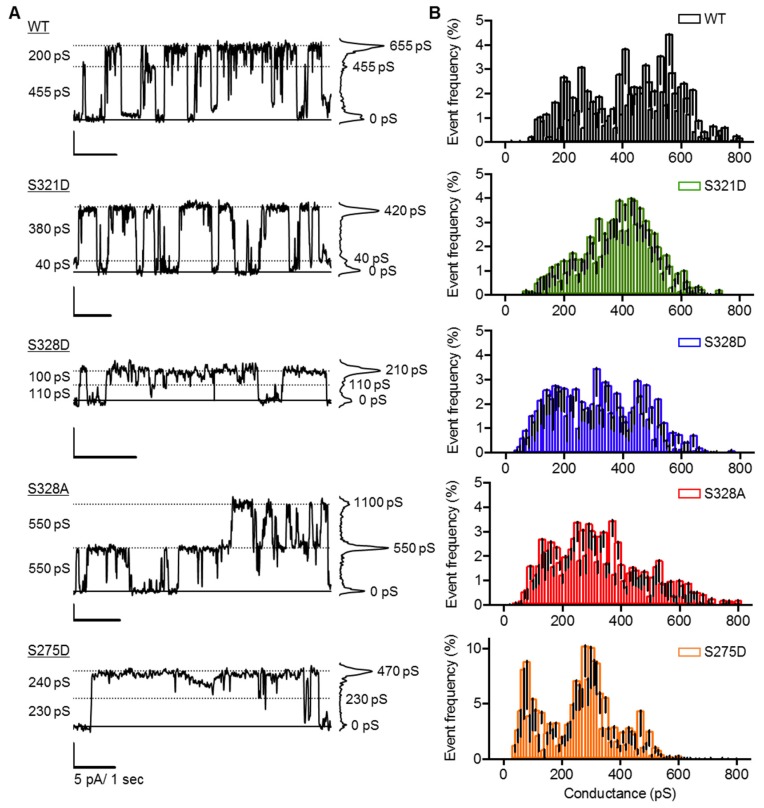
Cx37 (de)phospho-isoforms form HChs that transition between multiple open states. (**A**) Representative current traces and all associated points histogram with visible HCh transitions of variable size recorded from isolated cells in the presence of 1 mM [Ca^2+^]. V_m_= +25 mV for all traces. (**B**) HCh transition amplitude histograms of Cx37-isoforms. Cx37-WT: *N* = 16, *n* = 1044; -S321D: *N* = 24, *n* = 1943; -S328D: *N* = 8, *n* = 1620; -S328A: *N* = 11, *n* = 1692; -S275D: *N* = 25, *n* = 1071. Note change in range of event frequency values for Cx37-S275D as HCh transitions were less frequent due to long dwell times in the closed state.

Distinct HCh openings of varying size were evident in cells expressing each Cx37 (de)phospho-isoform studied in the presence of 1 mM [Ca^2+^]_o_ (Figure 5A). Despite modifying the phosphorylation state of select serine residues in the Cx37-CT, Cx37-mutant HChs were still able to transition between conductance states across the entire conductance range (~100–800 pS) for a single Cx37-WT HCh (Figure 5B). While the relative frequency of specific HCh transition amplitudes is apparently modified by (de)phospho-mimicking mutations in the Cx37-CT (shifting towards smaller conductance transitions), there were no obvious peaks in the transition amplitude histogram in common between the death-inducing Cx37 mutants, demonstrating that HCh conductance, alone, is not a predictive measure of a cell death phenotype. It is worth noting that unlike the other isoforms studied, Cx37-S275D HCh conformations were commonly stable for many seconds, therefore transitioning between conductance states infrequently and elevating the apparent frequency of events.

**Figure 6:**
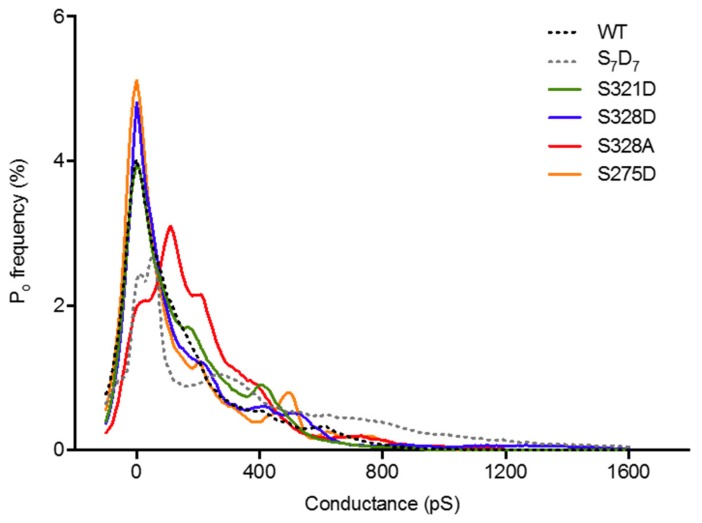
HCh P_o_ is not uniform between death-inducing mutants. Mean HCh P_o_ data plotted without error bars for ease of visualization. Cx37-WT: *N* = 8; -S321D: *N* = 11; -S328D: *N* = 8; -S328A: *N* = 6; -S275D: *N* = 8. Analyzed 240 s per cell with V_m_= +25 mV.

We also examined HCh P_o_, as previous data suggested that increased P_o_ of large conductance HChs may be predictive of cell death [22]. Figure 6 shows P_o_ for all conductance levels for each death-inducing mutant and Cx37-WT. There are clear differences between mutants (see ANOVA results in Table S1), but no conductance state is preferred by all death-inducing isoforms compared to Cx37-WT. The data suggest that while increased P_o_ of large conductance HChs may be sufficient to induce death, as with the Cx37-S_7_D_7_ or -S328A mutants, such HCh behavior is not necessary for Cx37-induced cell death.

Together, the HCh incidence, conductance, and P_o_ data indicate that there is no unique feature of HCh activity (detectable as current carrying differences) that distinguishes death-inducing mutants from non-death-inducing mutants, possibly suggesting that Cx37 can induce cell death in multiple ways.

### 2.5. Channel Function Is Necessary for pCx37-Induced Cell Death

**Figure 7:**
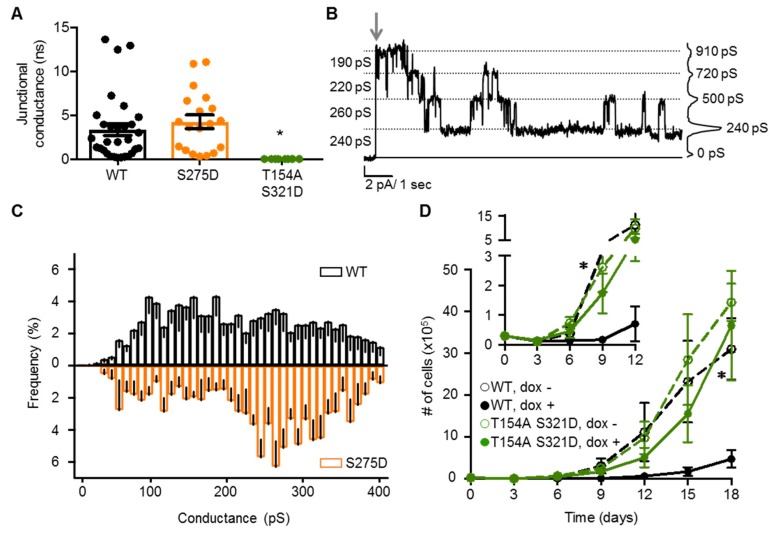
Channel functionality is necessary for Cx37-mediated cell death. (**A**) Cx37-WT and -S275D form functional GJChs with comparable junctional conductance. No evidence of coupling was apparent between cells expressing Cx37-T154A-S321D. Cx37-WT: 3.38 ± 0.7 nS, *N* = 30; -S275D: 4.25 ± 0.8 nS, *N* = 19; -T154A S321D: 0 nS, *N* = 8. * *p* < 0.05 vs WT. (**B**) Representative current trace of Cx37-S275D comprised GJChs showing a high frequency of 220–260 pS transition events. Grey arrow: start of 25 mV pulse. (**C**) Transition amplitude frequency plots for Cx37-WT (black) and -S275D (orange) GJChs suggest an increased preference for large amplitude transitions in iRin37-S275D cells. Cx37-WT *N* = 11, *n* = 1235; -S275D *N* = 6, *n* = 596. (**D**) Despite expression (dox +), iRin37-T154A-S321D cells (green) proliferate. *n* = 4 in triplicate for each isoform. Inset: Cx37-T154A-S321D eliminates the induced cell death and growth arrest characteristic of Cx37-WT expressing cells. All values are mean ± s.e.m (where error bars are not evident, they are smaller than the symbol size). *p* < 0.05 for dox + versus dox − for WT (∗) but not -T154A-S321D.

The density-dependence of Cx37-S275D’s death-inducing properties suggested that GJCh formation/function may be protective, as there was no cell death evident in contact-forming iRin37-S275D cells (when the ratio of GJCh:HCh is increased compared to cells at low density; Figure 4B). As such, we investigated GJCh function in iRin37-S275D cell pairs. Cx37-S275D supported a junctional conductance of 4.25 ± 0.8 nS (*N* = 19), comparable to Cx37-WT (Figure 7A). Multiple GJChs (3 or more) were typically active in iRin37-S275D cells, but they transitioned in 200–240 pS steps, evident in Figure 7B. Transitions in fairly regular intervals of ~250pS were noticeable in many GJCh records and the relative frequency amplitude histograms revealed the presence of these events (Figure 7C). Since all records contained more than one active Cx37-S275D GJCh, analysis of P_o_ was not possible. Nevertheless, when GJCh activity was present, the current level rarely returned to baseline (unless triggered by the addition of halothane), indicating that at least one channel (or several in a stable subconductance state) remained open when a transjunctional voltage was applied.

Given that the current carrying properties (conductance or P_o_) of neither HChs nor GJChs could explain all facets of pCx37-induced cell death, we determined whether pCx37 induces cell death independent of channel function. A double mutant including T154A, which renders both GJChs and HChs non-functional [23], and S321D, which induces cell death, was generated and expressed in iRin cells. Cell pairs expressing Cx37-T154A-S321D showed no evidence of coupling (Figure 7A), and proliferated at a rate comparable to non-expressing cells, without any apparent cell death or growth arrest prior to proliferation (Figure 7D). Collectively, these data suggest that while Cx37-mediated cell death requires a functional pore domain, non-channel functions of Cx37 likely contribute to cell death since GJCh and HCh conductance properties do not predict Cx37-mediated cell death. 

## 3. Discussion

Cxs are well-documented regulators of coordinated tissue function, including controlled growth. Cell death is a powerful inhibitor of proliferation and a large body of evidence suggests a role for Cx proteins in regulating the balance between the life and death of a variety of cell types during development [31,32], in disease (for example, cardiovascular [33] or cancer [34]), or following injury [35]. Most cell types express multiple Cxs, which makes it challenging to attribute a specific phenotypic change (between growth arrested, proliferative, and death states) to a specific Cx isoform [32]. Nevertheless, widespread apoptosis was evident by embryonic day 9.5 in animals deficient in Cx45 [36]. Fang and colleagues [37] showed that in the developing retina, Cx37 limits vessel pruning, a process relying on apoptosis [38]. Fang and colleagues [39] also showed that prolonged limb ischemia (induced by femoral-saphenous artery-vein pair resection) results in distal limb necrosis in WT, but not Cx37^-/-^ animals, suggesting that the absence of Cx37 limits injury-induced cell death. Cx43, the most ubiquitously expressed and widely studied Cx, has also been shown to participate in cell survival decisions during development, disease, and injury. Exposure of retinal endothelial cells to high glucose decreased gap junction-mediated intercellular communication and down regulated mitochondrial Cx43 expression, leading to cytochrome C release and cell death [40], phenomena observed in diabetic retinopathy [41]. Moreover, blocking Cx43 HChs with mimetic peptides reduces the extent of injury in diabetic retinopathy [35,42], suggesting such peptides as a promising therapeutic strategy for the treatment of this and other diseases. These, and many other studies, highlight the importance of Cxs in regulating the balance between the life and death of various cell types in physiological and pathophysiological settings. Importantly, while the mechanism(s) underlying cell survival regulation by Cxs remain uncertain, differential phosphorylation is key to Cx- and cell type-specific differences in phenotypic switching [22,43,44].

As the available data on Cx-dependent control of cell survival are diverse and complex, albeit sometimes contradictory, they suggest tissue- and Cx-specific regulation. Multiple mechanisms have been proposed over the last two decades regarding the regulation of cell survival and proliferation: intercellular communication (GJChs), transmembrane signaling (HChs), and channel-independent pathways (protein-protein interactions). Data presented in the current study indicate that Cx37-dependent cell death requires a pore domain able to function in a manner regulated by specific CT-phosphorylation, which suggests that, as discussed next, a combination of channel-dependent and -independent mechanisms regulate the cell growth phenotype.

Available data for phosphorylation-dependent regulation of Cx function primarily focus on one or a small number of residues and their impact on one molecular function. However, recent proteomic studies challenge the hypothesis of an “on/off” response for a variety of Cx functions by demonstrating that Cxs undergo multiple levels of multi-site phosphorylation (for a review, see [45]). This raises the possibility that Cx phosphorylation may work in a cooperative manner. As Cx37 contains 54 Ser/Thr/Tyr residues, many of which are located in the CT and are putative targets of closely related growth factor activated kinases, it is important to consider that there are more than 10^16^ phosphorylation-based signaling combinations within a single Cx37 subunit, let alone a given HCh (with six subunits) or even GJCh (with 12 subunits). Previous structural studies indicate that the Cx37-CT is predominantly unstructured [24,46]; an intrinsically disordered secondary structure is now recognized as a center for the regulation of protein function as the conformation of these domains may be dynamically modified by the local environment, phosphorylation, and/or interaction with binding partners [47]. Therefore, the Cx37-CT may be a highly flexible sensor that is capable of monitoring the microenvironment within the cell and serves as a hub for cell survival/growth signaling cascades.

Nuclear magnetic resonance (NMR) analysis of Cx43 suggests that there are two regions of regulatory control in the CT: aa 260–290 and the last 20 aa [48,49]. Both of these regions are highly enriched with kinase consensus sequences and interacting protein binding sites. Interestingly, NMR studies have shown that modifications in either of these regions result in conformational changes of the other region. For example, aspartate substitution to mimic phosphoserine at Cx43-S365 induces long-range structural changes of residues 275–286, suggesting intra-domain interaction of the Cx43-CT. The data presented herein would suggest a similar mechanism for Cx37, i.e., phosphorylation-sensitive interaction between the end-tail and mid-tail regions; shortening the Cx37-CT by deletion of either the end-tail or mid-tail region resulted in widespread cell death, but removal of the entire CT (both the end-tail and mid-tail domains) eliminated Cx37-dependent cell death [24]. That the death-inducing phenotype of Cx37-S328A is eliminated when alanine is also substituted at S275 and S302 (Cx37-S_3_A_3_) or at serines 275, 285, 302, 319, 321, and 325 (Cx37-S_7_A_7_; [22]) further implies intra-domain interaction. Therefore, the ability to switch phenotypes and overcome the death-inducing properties of Cx37 likely involves dynamic feedback from the end-tail to the mid-tail region, and vice versa. That is, phosphorylation within the end-tail domain may impact the phosphorylation state and/or potential binding partners of the mid-tail region to modify Cx37 function and induce cell death. 

That most iRin37-WT cells survive after induced expression suggests that either phosphorylation is not uniform across all cells or that the Cx37 phosphorylation state that induces death occurs transiently and most cells quickly adapt by modifying the phosphorylation state to something more amenable to survival. Mass spectrometry data and the expression of Cx37-isoforms that mimic constitutive (de)phosphorylation from the current study support this idea; the low incidence of phosphorylation at serine residues 275, 321, and 328 and the corresponding (de)phosphomimetic isoforms are consistent with the dynamic nature of Cx37-CT phosphorylation to induce cell death. Highlighted by the Cx37-S328 mutants, the inability to switch between phospho-states may be the stimulus for cell death. The hydroxyl group of serine 328 appears to be critical for Cx37-dependent survival and temporary, but not constitutive, phosphorylation at Cx37-S328 can support normal cell function. 

In contrast to the density-independent effects of Cx37-S321D, -S328D, and -S328A on cell survival, cell-cell contact rescued cells from Cx37-S275D-induced cell death suggesting dichotomous roles of HChs and GJChs in Cx37-mediated cell death. Two possible explanations for the density-dependent effects of Cx37-S275D are: (1) the balance between the passage of unidentified pro-survival factors from neighboring cells through GJChs and the simultaneous depletion of pro-survival factors from inside the cell to the extracellular space through HChs modulate the magnitude of cell death; and (2) the phosphorylation state of the Cx37-CT at sites other than Cx37-S275 changes with cell-cell contact (i.e., phosphorylation states of GJChs and HChs are not identical and therefore the growth phenotype changes with cell-cell contact and GJCh formation).

As for the mechanism underlying Cx37-induced cell death, we showed, previously and here, that Cx37 HCh and GJCh function (conductance and P_o_) are modulated by phosphorylation in the CT, and suggested [22] that increased P_o_ of fully open channels might be predictive of cell death. Despite a similar outcome of cell death (including at least some cells by apoptosis), the noticeable differences in the channel current carrying capacity suggest that an increased preference for large conductance open states is sufficient, but not necessary, for Cx37-induced cell death. It is possible that another functional parameter (e.g., increased permeability to common cell death mediators such as Ca^2+^ or IP_3_) is responsible for Cx37-mediated cell death, although this remains to be determined. Alternatively, a phosphorylation-sensitive but channel-independent function of Cx37 may induce cell death. It was previously shown that the PKC target site S262 regulates Cx43-mediated inhibition of DNA synthesis of cultured myocytes at a higher density, but not in the absence of cell-cell contact [50]. The authors suggested that the proteins interacting with Cx43-S262 may be different, depending on cell-cell contact, and that S262 may only interfere with these interactions in non-contact forming cells. As a pore domain able to function and phosphorylation-specific intra-domain interactions are necessary for Cx37-mediated cell death, a combination of the aforementioned mechanisms may be responsible. Results from iRin37-T154A-S321D cells reinforce and expand on these conclusions. 

Altogether, the data suggest that phosphorylation-specific conformations (as mimicked by alanine and aspartate substitutions) of the channel and CT domains regulate Cx37-induced cell death. Specifically, the phosphorylation-sensitive interaction between the end-tail and mid-tail regions of the CT is critical for cell survival and phenotypic switching. While the mechanism(s) of Cx37 death induction remain to be fully understood, we speculate that the use of tumor-specific promoters and lenti- or adeno-viral vectors for the expression of these death-inducing isoforms of Cx37 could be used to seek out and kill tumor cells. 

## 4. Materials and Methods 

### 4.1. Cell Culture

Rin 1046 cells (provided by Dr. Ronald Lynch, University of Arizona) transfected with pTET-ON (Clontech, Mountain View, CA, USA) to create the iRin cell line were maintained in RPMI 1640 medium (Sigma Aldrich, St. Louis, MO, USA) supplemented with 10% Fetal Plex serum (Gemini Bio Products, West Sacramento, CA, USA), 300 µg/mL G418 (Life Technologies, Grand Island, NY, USA), 300 µg/mL penicillin (Sigma Aldrich), and 500 µg/mL streptomycin (Sigma Aldrich). Cells were maintained at 37 ºC in a humidified, 5% CO_2_ incubator. iRin cells transfected to inducibly express an isoform of mCx37, for example iRin37-WT, were maintained in 100 µg/mL hygromycin (Life Technologies) and Cx37 expression was initiated with doxycycline (dox).

### 4.2. Mutant Plasmid Generation, Transfection, and Cloning

All deletions and/or point mutations were introduced into the pTRE2h-mCx37 plasmid using properly designed primers (Table S2; Operon Biotechnologies, Huntsville, AL, USA). A V5-tag was added to deletion mutants for ease of detection and protein expression. The plasmid containing the mid-tail (aa 273–317) deletion was made in a three-step amplification process: (1) aa 1–272 and 318–323 were linked with primers I & II and full length Cx37 in the pTre2-Hygro plasmid (Figure S4A,B); (2) aa 269–272 were linked to aa 318–333, and the V5 tag and *NheI* restriction site were linked with primers III & IV (Figure S4C,D); and (3) PCR products from steps 1 and 2 were combined using primers I & IV to make the contiguous Cx37-dM (containing aa 1–272 + 318–333) + V5 plus the 5’ *BamHI* and 3’ *NheI* restriction sites (Figure S4E,F). The resulting plasmid was directionally cloned into *BamHI* and *NheI* sites in pTRE2h. For the end-tail deletion (truncation after aa 317), two primers were used to introduce a V5 tag and a stop codon at position 318: 5’ pTRE2 forward primer and 3’ primer containing a partial Cx37 sequence, V5-epitope, a stop codon, and an *NheI* restriction site. The mutant sequence was PCR amplified and purified per the manufacturer’s instructions and subsequently ligated into pTRE2h. Single- and multi-site mutations were introduced using the QuikChange Site-Directed Mutagenesis or QuikChange Multi Site-Directed Mutagenesis Kits (Agilent Technologies, La Jolla, CA, USA) and appropriate primers (Table S2). All sequences were confirmed at the University of Arizona Genomics Core Sequencing Facility and the mutant plasmids were transfected into iRin cells using Lipofectamine 2000 (ThermoFisher Scientific; San Jose, CA, USA), according to the manufacturer’s instructions. Cells resistant to hygromycin were isolated and dilution cloned. Clones were screened for maximum Cx37 expression by Western blotting after 24 and 48 h exposure to 1, 2, and 4 µg/mL doxycycline (dox). For 19 different Cx37 variants, this strategy resulted in 9.4 ± 2.2 fmoles/µg total cell protein with each phenotype (proliferative, growth arrest, death) observed throughout the range of expression (0.27–40 fmole/µg total cell protein). Maximal induction of protein expression was initiated by the addition of 2 µg/mL dox for iRin37-WT, -dM, -dMA_4_, -dMD_4_, -dE, -dEA_3_, -dED_3_, and -S275A; 1 µg/mL for iRin37-S328D; and 4 µg/mL for iRin37-S328A, -S275D, -S321D, -T154A S321D, and -S321A. Importantly, in the absence of dox, no Cx37 could be detected.

### 4.3. Immunoblotting

The whole cell protein was isolated as previously described [25], the protein content was assessed using the Pierce BCA protein assay (Life Technologies), and 20–50 μg of sample protein was loaded onto precast 12% SDS-PAGE gels (Bio-Rad, Hercules, CA, USA). After being transferred to nitrocellulose, blots were blocked with 5% non-fat dry milk and Cx37 detected with anti Cx37-18264 ([51]; a gift from Dr. Alexander Simon, University of Arizona), anti-V5 (ThermoFisher Scientific), or anti-caspase 3 (Cell Signaling, Danvers, MA, USA). Primary antibodies were visualized using enhanced chemiluminescence strategies with SuperSignal West Dura or Femto Systems (Thermo Scientific, Waltham, MA, USA) and an anti-rabbit (for Cx37 and caspase 3; GE Lifescience, Pittsburg, PA, USA) or anti-mouse (for V5; Promega; Madison, WI, USA) HRP-conjugated secondary antibody using a Kodak Image Station 2000 or Syngene Gbox Chemi-Xr5.

### 4.4. Mass Spectrometry

Cells were grown to ~80% confluence in 150 mm plates and treated with 1µg/mL dox for 24 h to induce Cx37-WT protein expression and growth arrest of the majority of the cells. In some experiments, cells were treated with 0.2 µM bisindolylmaleamide (BIM, from DMSO stock, a specific PKC inhibitor) for 60 min at 37 °C or 50 ng/mL 12-O-tetradecanoylphorbol-13-acetate (TPA, from ethanol stock, a non-specific PKC agonist) for 30 min at 37 °C. Plates were rinsed three times in cold PBS and cells were then lysed in 400 µL buffer (25 mM Tris, pH 7.4, 100 mM NaCl, 10 mM EDTA, 0.5% Triton-X100 in PBS, 0.6% SDS, 50 mM NaF, 0.5 mM Na_3_VO_4_, 2 mM PMSF in ethanol, 1× protease inhibitor cocktail (Roche, Basel, Switzerland)). Lysed cells were scraped into a 2 mL microcentrifuge tube and sonicated (two 10-s pulses). A total of 400 µL of lysis buffer without SDS was added and tubes were rocked at 4 °C for 20 min to ensure complete lysis. Lysates were spun at 10,000 *g* for 15 min at 4 °C to pellet any remaining cell debris and the supernatant was moved to clean 1.5 mL tubes. To preclear lysates, 45 µL of Protein A/G sepharose beads (Promega) was added for 30 min of rocking at 4 °C. Beads were pelleted at 2000 *g* for 2 min and the supernatant was moved to a clean 1.5 mL tube. A total of 45 µL of beads and 5 µg of antibody (anti-V5: ThermoFisher; anti-Cx37: Cx37-18264 [51]) were added to precleared lysates and rocked overnight at 4 °C. Beads were pelleted at 2000 *g* for 2 min and washed three times with 1 mL of PBS. Following the final wash, a gel-loading tip was used to remove any remaining wash buffer without disturbing the beads. Beads were then resuspended in 10 µL of sample buffer (125 mM Tris, 4% SDS, 20% glycerol, 0.004% bromophenol blue, 5% β-mercaptoethanol and 0.02% NaN_3_), prior to heating for 5 min at 100 °C and loading onto a 12% TGX gel (Biorad). Gel was run in Tris-glycine running buffer (190 mM glycine, 1% SDS, 25 mM Tris) for 10 min at 125 V and 35 min for 180 V.

Gels were stained with Bio-Safe Coomassie G-250 Stain and the single band corresponding to the location of Cx37 on the SDS-PAGE gel was excised. The gel slices were subjected to trypsin digestion and the resulting peptides were purified by C18-based desalting exactly as previously described [52]. HPLC-ESI-MS/MS was performed in positive ion mode on a Thermo Scientific Orbitrap Fusion Lumos tribrid mass spectrometer fitted with an EASY-Spray Source (Thermo Scientific). NanoLC was performed using a Thermo Scientific UltiMate 3000 RSLCnano System with an EASY Spray C18 LC column (Thermo Scientific, 50 cm × 75 μm inner diameter, packed with PepMap RSLC C18 material, 2 µm, cat. # ES803); loading phase for 15 min at 0.300 µL/min; mobile phase, linear gradient of 1–34% Buffer B in 119 min at 0.220 µL/min, followed by a step to 95% Buffer B over 4 min at 0.220 µL/min, hold 5 min at 0.250 µL/min, and then a step to 1% Buffer B over 5 min at 0.250 µL/min and a final hold for 10 min (total run 159 min); Buffer A = 0.1% FA/H2O; Buffer B = 0.1% FA in 80% ACN. All solvents were liquid chromatography mass spectrometry grade. Spectra were acquired using XCalibur, version 2.3 (Thermo Scientific). A “top speed” data-dependent MS/MS analysis was performed. Dynamic exclusion was enabled with a repeat count of 1, a repeat duration of 30 s, and an exclusion duration of 60 s. Tandem mass spectra were extracted from Xcalibur ‘RAW’ files and charge states were assigned using the ProteoWizard 3.0.1 msConvert script employing the default parameters [53]. The fragment mass spectra were then searched against the mouse SwissProt_2016_10 database (23,550 entries) using Mascot (Matrix Science, London, UK; version 2.4) employing the default probability cut-off score. The search variables that were used were: 10 ppm mass tolerance for precursor ion masses and 0.5 Da for product ion masses; digestion with trypsin; a maximum of two missed tryptic cleavages; variable modifications of oxidation of methionine and phosphorylation of serine, threonine, and tyrosine. Cross-correlation of Mascot search results with X! Tandem was accomplished with Scaffold (version Scaffold_4.8.7; Proteome Software, Portland, OR, USA). Probability assessment of peptide assignments and protein identifications were made through the use of Scaffold. Only peptides with ≥95% probability were considered. The mass spectrometry proteomics data have been deposited at the ProteomeXchange Consortium via the PRIDE partner repository [54] with the identifier (PDX012191). 

### 4.5. Proliferation

Cells were plated at low density (3 × 10^4^ cells/well; ~3125 cells/cm^2^) in triplicate in six-well plates designated for each collection time point. Additionally, 24 h after plating, day zero in proliferation assays, dox was added to the dox + wells; dox was refreshed every other day thereafter for the duration of the experiment. Every three days, cells were lifted and counted using a Cellometer (Nexcelom, Lawrence, MA, USA). In some experiments, cells were seeded at low density and maintained for 12–14 days in the absence of dox, after which dox was introduced to assess the impact of cell density (i.e., cell-cell contact, see Figure S1) on Cx37-induced cell death.

### 4.6. Electrophysiology

To characterize GJCh and HCh activity, cells were plated at low density on glass coverslips and dox was added to induce maximal protein expression. Additionally, 24 h later (or 48 h if no functional channels were detected at 24 h), coverslips were mounted in a custom-made chamber and recordings made on attached, healthy appearing cells. Cells were immersed in an external solution containing (in mMol/L): 142.5 NaCl, 4 KCl, 1 MgCl_2_, 5 glucose, 2 Na Pyruvate, 10 HEPES, 15 CsCl, 10 TEA-Cl, 1 CaCl_2_, pH adjusted to 7.2, 315 mOsM. Patch pipets (3–10 MΩ) contained (in mMol/L): 124 KCl, 14 CsCl, 9 HEPES, 9 EGTA, 0.5 CaCl_2_, 5 glucose, 9 TEA-Cl, 3 MgCl_2_, 5 Na_2_ATP, pH 7.2, 315 mOsM. All channel activity was recorded from cells within an hour of being placed in warmed external solution.

#### 4.6.1. GJCh Electrophysiology

Junctional conductance (g_j_) was determined as previously described [25,55] using dual whole-cell voltage-clamp techniques with Axopatch1D amplifiers and pClamp software (Molecular Devices, Sunnyvale, CA, USA). g_j_ was assessed with 10 mV transjunctional pulses delivered every 30 s. Single channel activity was recorded using a transjunctional voltage of 25 mV; halothane was used, as necessary, to reduce g_j_ below 1 nS, but never in experiments assessing channel P_o_. Current records were digitized at 2.5 kHz and filtered at 50 Hz, and the amplitudes of current transitions (events, ‘*n*’) in the record, where current levels before and after the transition were stable for greater than 50 ms, were measured. Event amplitudes were converted to conductances (pA/25 mV), events grouped into 10 pS bins, and the number of events in each bin relative to total events for each cell pair calculated. Mean data for each bin from multiple cell pairs (‘*N*’) collected on multiple days was then determined and plotted as a relative frequency histogram. For event frequency plots, the y-axis displays positive values in both directions as a convenient method of comparison between genotypes (if there were no differences, these plots would appear as mirror images of each other). Displayed current traces include the corresponding all-digitized-points histograms for the trace. 

#### 4.6.2. HCh Electrophysiology

HCh activity was assessed using the same general approach as for GJChs, with +25 mV transmembrane voltage pulses applied to single isolated cells. Event amplitudes were measured and plotted as described for GJChs. P_o_ was determined from current records in which the pulse protocol included an initial ~30 s pulse to +25 mV followed by ~2 s at 0 mV and a second pulse to +25 mV of ~4.5 min duration. Additionally, 240 s of the decimated, filtered, second pulse were compiled into 0.25 pA bins (spanning from −4 to 100 pA), the relative frequency of digitized points (each 0.002s) was calculated for each bin, and frequency versus current was plotted. The current level associated with the center of the first peak (determined visually or following peak fitting with Origin software) was assumed to represent the closed state current, which was subtracted from all current bins, thereby defining the 0 pA (closed state) bin. Data from multiple records were aligned at the closed state bin, and the average relative frequency for each current bin across cells (‘*N*’) was determined. The average relative frequencies for each Cx37 isoform were plotted as a function of conductance. Isoforms were analyzed for differences (ANOVA and Tukey’s multiple comparisons test using Prism software) in frequency (P_o_) in the −2.5–2.5 pA, 2.5–7.5 pA, 7.5–12.5 pA, and >12.5 pA, which correspond to the closed state, 100–300 pS, 300–500 pS, and >500 pS conductance levels. 

### 4.7. Fluorescence Activated Cell Sorting

Cells were plated in increasing amounts (0.5−4 × 10^6^ cells)/100 mm dish. Cx37 protein expression was induced 24 h later with the addition of dox. Dox was replenished every 48 h for the duration of the experiment. After six days of protein expression, adherent and non-adherent cells were harvested, counted separately using a Cellometer, pooled, and fixed in 70% EtOH. After repelleting, the EtOH was removed, and 10^6^ cells were resuspended in 0.5 mL PBS containing 50 µg RNase A. Cells were stained with 50 µg propidium iodide as a measure of DNA content and 10^4^ events (cells) were analyzed on a FACS CantoII at the UAGC/ARL Cytometry Core Facility. Cell cycle distribution was determined using ModFit-LT software with doublet discrimination. Debris/dead cells were identified as events that contained less than a full complement of DNA and expressed as a percentage of 10^4^ total analyzed events.

### 4.8. Statistics

For proliferation curves, the slopes of logarithm transformed dox + vs. dox − data were compared; for all mutants, p values less than 0.02 indicated the dox + and dox − data were significantly different. For the dox switch experiments, where cells were grown 12–14 days without dox followed by 7–9 days with dox, the slopes of logarithm transformed pre- and post-dox addition were compared. Comparisons of proliferation curves across mutants were, in general, not done because differences in protein expression levels, number and insertion site(s) of gene copies, and mechanism of death induction could contribute, along with the introduced mutation, to the timing and extent of induced death. Where comparisons of growth/death across mutants were performed, a non-parametric ANOVA with Kruskal-Wallis multiple comparisons test was used. Junctional conductance measurements were analyzed by unpaired, two tailed, Student’s t-tests. Chi-squared and Fisher’s exact tests were used to analyze HCh incidence. ANOVA with Tukey’s post-hoc test was used to analyze HCh P_o_ differences. Significant differences were noted as those with *p* < 0.05. Error bars represent mean ± s.e.m.

## 5. Conclusions

**Figure 8:**
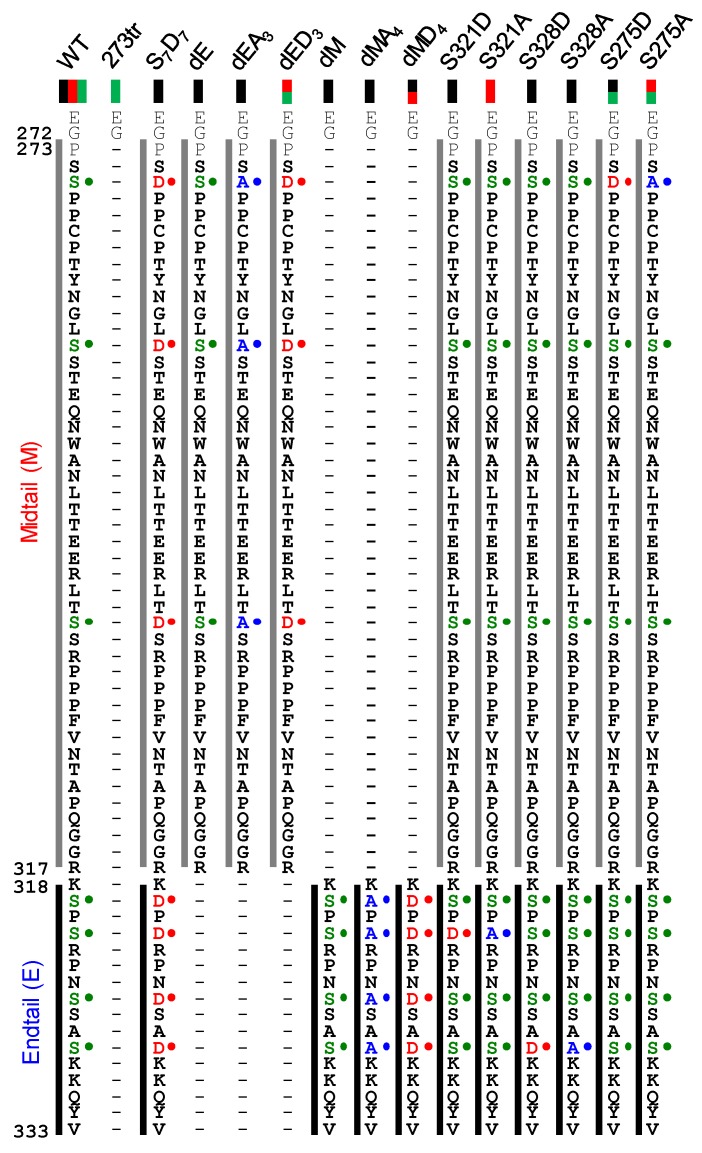
Primary sequence alignment of Cx37 isoforms denoting deletions (-), point mutations (•), and the associated growth phenotypes (colored rectangles beneath mutant titles; black: cell death, red: growth arrest, green: proliferation) of each isoform when expressed in iRin cells. The mid-tail (M) region spans from P273 to R317. The terminal 16 amino acids (318–333) constitute the end-tail (E) region. For additional data pertaining to Cx37-WT, -273tr, and -S_7_D_7_ mutants, see [22,24,25].

Altogether, the data suggest the phosphorylation-dependent regulation of Cx37-induced cell death. As multiple isoforms of Cx37 (with deletions, single and multi-site point mutations mimicking (de)phosphorylated serine residues, Figure 8) induce cell death without unifying properties (density-dependent and -independent cell death, HCh and/or GJCh current carrying properties), the data imply that Cx37 mediates cell death using multiple strategies, likely including a combination of channel-dependent and -independent mechanisms.

## Figures and Tables

**Table 1:** Identification of Cx37 phosphorylation sites in iRin37 cells by tandem mass spectrometry.

Site	Detected	Phosphorylated ^1^
S275	33	5
S285	57	0
S302	559	0
S319	559	204
S321	559	4
S325	559	2
S328	559	6

^1^ Also detected as phosphorylated: T154 (4), Y177 (10), T280 (1), T287 (27), and T311 (4). Data from 37 samples, with a total of 2234 analyzed peptides. See link in Material and Methods for complete set of mass spectrometry data.

## Supplementary Materials

The following are available online at http://www.mdpi.com/2072-6694/11/2/188/s1, Figure S1: Cell density and cell-cell contact increase as a function of time post-plating in the absence of Cx37 expression (dox -). Figure S2: Cx37-S321A and Cx37-S275A do not induce death when expressed in iRin cells. Figure S3: Activated caspase 3 is detected in non-adherent, dying cells induced to express Cx37-S321D, -S328D, -S328A, or -S275D. Figure S4: Schematic of strategy used to generate the Cx37-dM construct. Table S1: Significance of differences in HCh P_o_ between Cx37-WT and (de)phospho-mimicking mutants. Table S2: List of primers used to generate Cx37 mutants.
