# Supplementary material for: Phosphorylation-Dependent Intra-Domain Interaction of the Cx37 Carboxyl-Terminus Controls Cell Survival
Source: Cancers (Basel). 2019 Feb 6;11(2):188. doi: 10.3390/cancers11020188 (PMC6406260; doi:10.3390/cancers11020188)
Supplement: Supplementary file 1 [file cancers-11-00188-s001.pdf]

Supplementary Materials: Phosphorylation-Dependent Intra-Domain Interaction of the Cx37 Carboxyl-Terminus Controls Cell Survival

Nicole L. Jacobsen, Tasha K. Pontifex, Paul R. Langlais, and Janis M. Burt

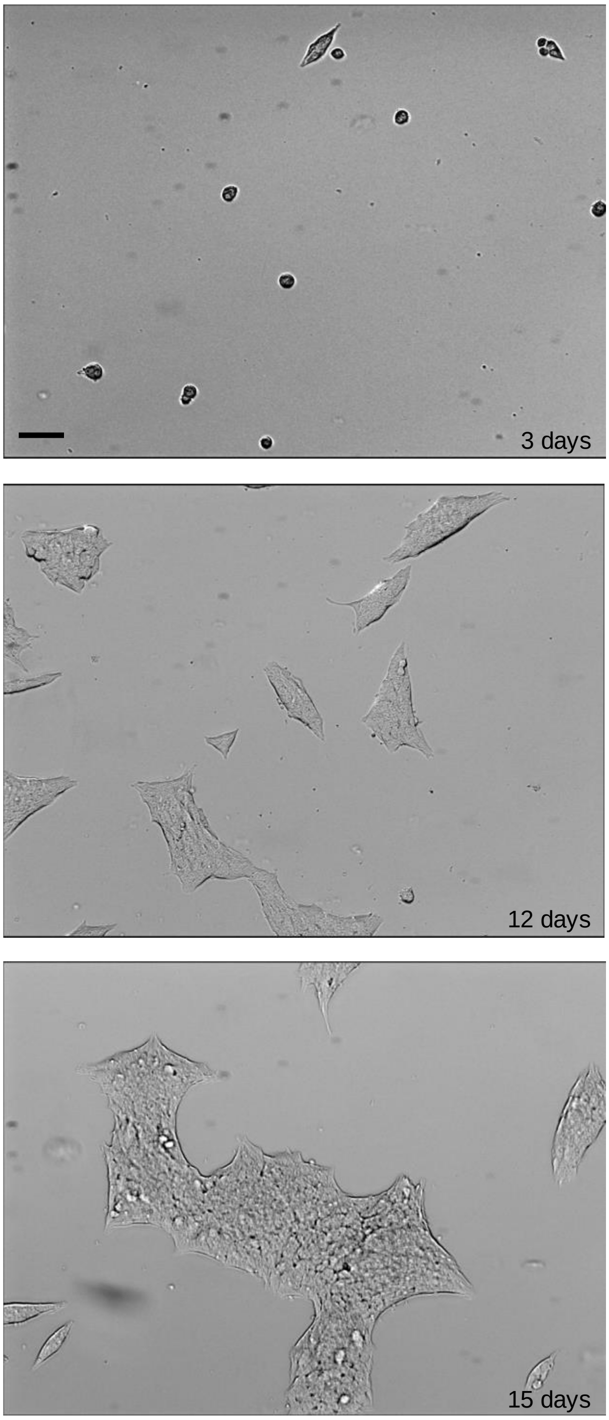

Figure S1. Cell density and cell-cell contact increase as a function of time post-plating in the absence of Cx37 expression (dox -). iRin37 cells plated at an initial density of 3,125 cells/cm^2 imaged at 3, 12 and 15 days. Scale bar applies to all images: 30µm.

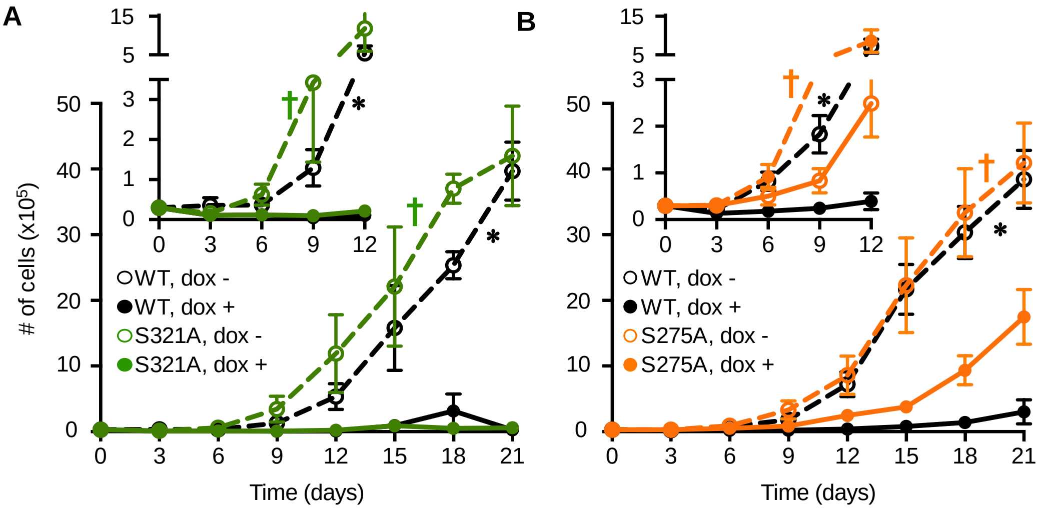

Figure S2. Cx37-S321A and Cx37-S275A do not induce death when expressed in iRin cells. **(A)** Expression of Cx37-S321A induces potent growth arrest at low density. **(B)** iRin37-S275A cells proliferate after a short period of growth arrest (days 0-3), little or no cell death occurs.

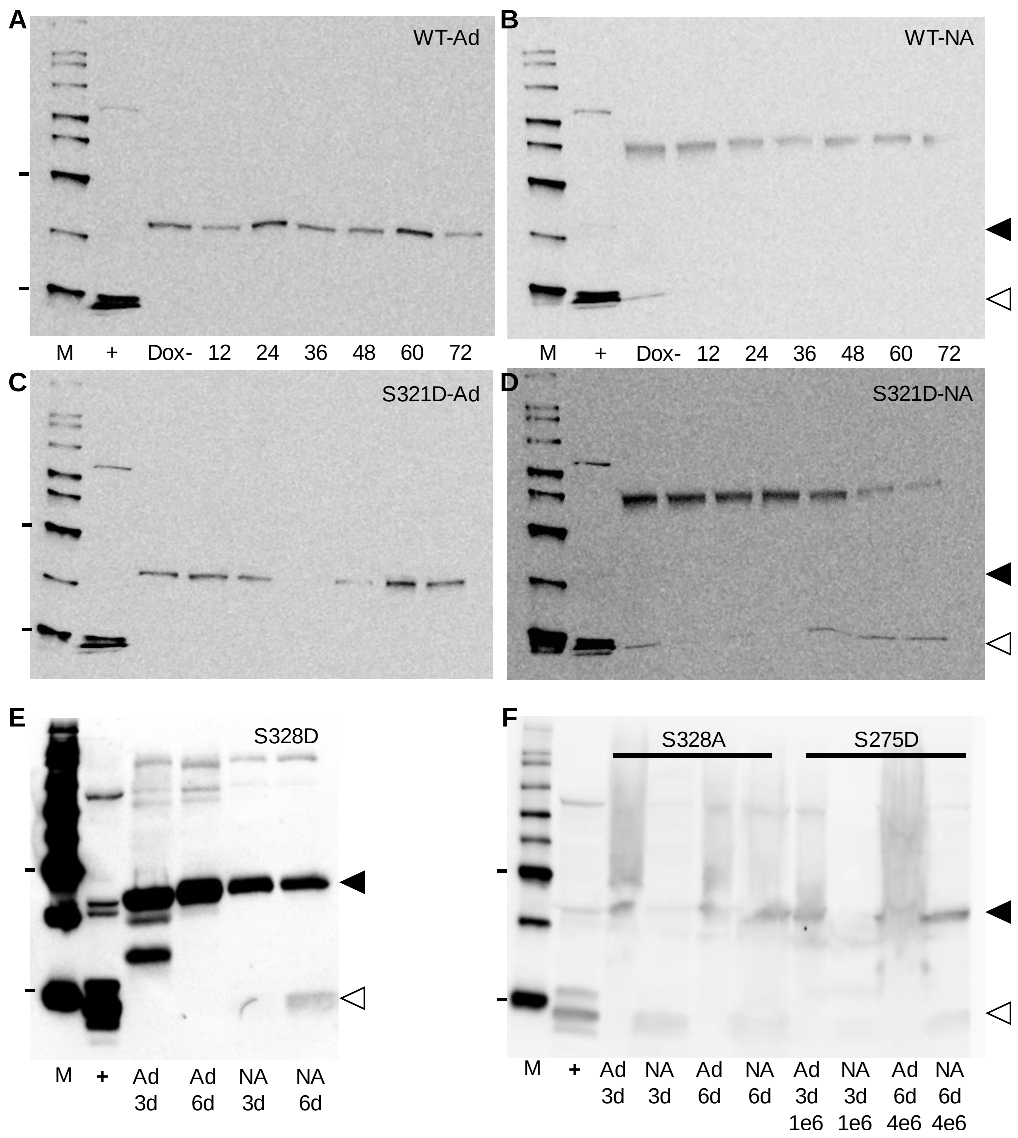

Figure S3. Activated caspase 3 is detected in non-adherent, dying cells expressing Cx37-S321D, -S328D, -S328A or -S275D. (**A**,**B**) Activated caspase 3 was not detected in the adherent

(Ad; still living) or non-adherent (NA; dying) Cx37-WT expressing cells. (**C,D**) Activated caspase 3 was detected in dying, NA cells but not in the adherent cells. In the Cx37-WT and -S321D blots adherent (Ad) and non-adherent (NA) cells were collected from non-induced cells (dox-) and cells induced with dox for 12, 24, 36, 48, 60 and 72 hours. (**E,F**) In S328D, S328A, S275D blots, Ad and NA cells were collected from cells induced for 3 or 6 days. For all blots, the left lane shows molecular mass markers (M) with the 40 and 20 kDa bands marked; the second lane (+) contains the positive control for activated caspase 3. Solid triangle shows the position of uncleaved caspase 3 and the open triangle shows the position of activated (cleaved) caspase 3.

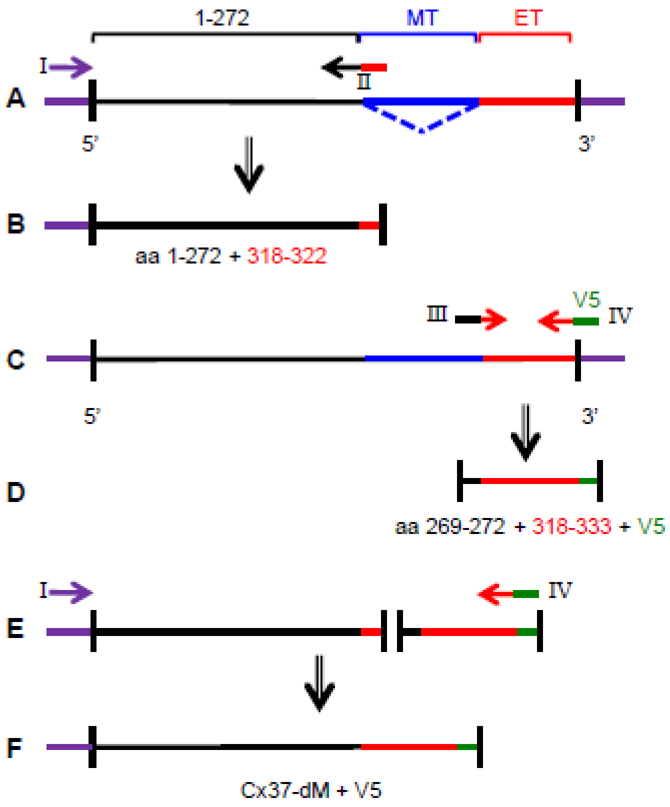

Figure 4. Schematic of strategy used to generate the Cx37-dM construct. The plasmid containing the mid-tail (aa 273-317) deletion was made in a 3-step amplification process. **(A,B)** aa 1-272 and 318-323 were linked with primers I & II and full length Cx37 in pTre2-Hygro plasmid. **(C,D)** aa 269-272 were linked to aa 318-333 plus V5 tag and *NheI* restriction site were linked with primers III & IV, **(E,F)** PCR products from steps 1 and 2 were combined using primers I & IV (E) to make the contiguous Cx37-dM (F; containing aa 1-272 + 318-333) + V5 and the 5' *BamHI* and 3' *NheI* restriction sites. The resulting plasmid was directionally cloned into *BamHI* and *NheI* sites in pTRE2h.

Table S1. Significance of differences in HCh P_o between Cx37-WT and (de)phospho-mimicking mutants. P values for the comparison of P_o for the isoform in the left column to remaining columns for each group; e.g. closed state probability of WT HChs is not different from S321D but significantly greater than the closed state probability of S328A. * indicates $p < 0.0001$; ns indicates not significant.

**WT**	S321D	S328D	S328A	S275D
Closed	ns	ns	>, *	ns
All open states	ns	ns	<,*	ns
100-300 pS	ns	ns	<,*	ns
300-500 pS	<, 0.0054	ns	<, 0.045	ns
500-800 pS	>, 0.0025	ns	ns	ns
>800 pS	ns	<,*	<, *	ns

**S321D**	S328D	S328A	S275D
Closed	ns	>, *	ns
All open states	ns	<, *	<, *
100-300 pS	ns	<,*	>, 0.0034
300-500 pS	ns	ns	>, 0.0233
500-800 pS	ns	<, *	<, 0.0004
>800 pS	<, *	>, *	ns

S328D	S328A	S275D
Closed	>, *	ns
All open states	<, *	ns
100-300 pS	<, *	ns
300-500 pS	<, 0.0424	ns
500-800 pS	ns	ns
>800 pS	ns	>, *

S328A	S275D
Closed	<, *
All open states	>, *
100-300 pS	>, *
300-500 pS	>, 0.0164
500-800 pS	ns
>800 pS	>, *

Table S2. List of primers used to generate Cx37 mutants.

**Construct/mutation**	**Primer sequence 5′ to 3′**
**1-272 + 318-323**	F (I): CGCCTGGAGACGCCATTCC (plasmid sequence)<br>R (II): GCTAGGGGACTTTCCCTCGCCCATGGGGAG
**269-272 + 318-333 + V5**	F (III): ATGGGCGAGGGAAAGTCCCCTAGCCGCCCC<br>R (IV): CTAGCTAGCCTACGTAGAATCGAGACCGAGGAGAGGGTTAGGGATAGGCTTACC CACATACTGCTTCTT (stop codon/restriction site)
**dM +V5**	F (I): CGCCTGGAGACGCCATTCC<br>R (IV): CTAGCTAGCCTACGTAGAATCGAGACCGAGGAGAGGGTTAGGGATAGGCTTACC CACATACTGCTTCTT
**dE +V5**	F (I): CGCCTGGAGACGCCATTCC<br>R: CTAGCTAGCCTACGTAGAATCGAGACCGAGGAGAGGGTTAGGGATAGGCTTACC CTTTCGGCCACCCTG
**S319A, S321A**	F: CCATGGGCGAGGGAAAGGCACCAGCCCGCCCCAACAGCTCTGC<br>R: GCAGAGCTGTTGGGGCGGGCTGGTGCCTTTCCCTCGCCCATGG
**S325A, S328A**	F: CGAGGGAAAGGCCCCTGCCCGCCCCAACGCCTCTGCAGCCAAGAAGCA<br>R: TGCTTCTTGGCTGCAGAGGCGTTGGGGCGGGCAGGGGCCTTTCCCTCG
**S319D, S321D**	F: CCCATGGGCGAGGGAAAGGATCCAGACCGCCCCAACAGCTCTGC<br>R: GCAGAGCTGTTGGGGCGGTCTGGATCCTTTCCCTCGCCCATGGG
**S325D, S328D**	F: CGAGGGAAAGGACCCTGACCGCCCCAACGACTCTGCAGACAAGAAGCA

	R:	TGCTTCTTGTCTGCAGAGTCGTTGGGGCGGTCAGGGTCCTTTCCCTCG
**S275A**	F:	GGGACCCTCTGCCCCACCGTGTC
	R:	GACACGGTGGGGCAGAGGGTCCC
**S302A**	F:	GAGAGACTGACCGCTTCCAGACTCCC
	R:	GGGAGGTCTGGAAGCGGTCAGTCTCTC
**S285A**	F:	CCTACAACGGGCTCGCTTCCACTGAGCAGAAC
	R:	GTTCTGCTCAGTGGAAGCGAGCCCGTTGTAGG
**S321A**	F:	GAAAGTCCCCTGCACGCCCCAACAG
	R:	CTGTTGGGGCGTGCAGGGGACTTTC
**S275D**	F:	CATGGGCGAGGGACCCTCTGATCCACCGTGTCCCACCTAC
	R:	GTAGGTGGGACACGGTGGATCAGAGGGTCCCTCGCCCATG
**S302D**	F:	CACAGAGGAGAGACTGACCGACTCCAGACCTCCCCCATTTG
	R:	CAAATGGGGGAGGTCTGGAGTCGGTCAGTCTCTCCTCTGTG
**S285D**	F:	CCTACAACGGGCTCGACTCCACTGAGCAGAAC
	R:	GTTCTGCTCAGTGGAGTCGAGCCCGTTGTAGG
**S321D**	F:	GCCGAAAGTCCCCTGATCGCCCCAACAGCTC
	R:	GAGCTGTTGGGGCGATCAGGGGACTTTCGGC
